# Supplementary material for: Action information is integrated into entorhinal representations of conceptual space and is reflected in eye movements
Source: PLoS Biol. 2026 Apr 13;24(4):e3003755. doi: 10.1371/journal.pbio.3003755 (PMC13089867; doi:10.1371/journal.pbio.3003755)
Supplement: S1 Appendix — Fig A. Test of noise in cross-validated RSA. Cross-validated neural distances within RDMs should be positive if the resulting matrix is driven by consistent pattern responses across runs. The mean distances were greater than zero in both entorhinal cortex ROIs, suggesting that ROI results were driven by signal rather than noise. Fig B. Reliability-based voxel selection. To establish if our entorhinal effects were robust to voxel-level noise, we carried out a permutation-based version of voxel reliability selection to select voxels which responded consistently to each condition across runs. Reduced ROIs were defined using these voxels, and the affordance effects persisted within them. Fig C. Comparison of affordance effect in right entorhinal cortex to noise ceiling. The affordance model correlations were compared to a noise ceiling estimated using across-participant variance. The effect in the right entorhinal cortex was not significantly below the estimated noise ceiling, suggesting another model could not have better explained our neural RDMs. Fig D. Affordance effect in right entorhinal cortex is not driven by task difficulty. Affordance effects remained significant after accounting for task difficulty by regressing out a model based on reaction time in probe trials. Fig E. Distance in visual space correlates with visual but not entorhinal cortex. (a,b) Neural pattern similarities in visual cortex were correlated with a model based on estimated gaze distance. This was not the case in entorhinal cortex. (c) Moreover, the affordance effect remained significant after controlling for gaze distance using partial correlation. Fig F. Eye movement differences in the y-axis. Participants showed a lateralized gaze effect in the y-axis as a function of affordance direction. Unlike in the x-axis, this effect was not correlated with performance. Fig G. Affordance representation in the motor cortex. The affordance model was also represented in the right motor cortex, possibly [file pbio.3003755.s001.pdf]

SUPPLEMENTARY MATERIALS

Test of noise in cross-validated RSA

As crossnobis is a cross-validated distance measure, we can also use the resulting similarity scores to assess how much a given region is consistently responsive to the conditions across runs. Namely, a score greater than 0 indicates a consistent response (Diedrichsen et al., 2016; Walther et al., 2016). To test this, for each region we calculated the mean distance within the neural RDM for each ROI and tested against 0 using a Wilcoxon signed rank test. Note that this is a conservative test, as we predict a distance of 0 for some state combinations in the affordance model.

Both regions showed mean distance values greater than 0 (Wilcoxon signed rank test, $p<0.0001$ for both ROIs), indicating the results were driven by signal (that is, consistent voxel patterns across runs) rather than noise.

It is worth noting, furthermore, that if only subjects with the mean distances greater than 0 are selected, the affordance RSA effect in the right entorhinal cortex becomes stronger ($n=35$, $t(34)=3.15$; $p=0.0046$ in a one-tailed t-test), whereas in the left entorhinal cortex this is not the case ($n=40$, $t(39)=-0.0090$; $p=0.50$). This further indicates that for subjects with consistent neural pattern responses between runs for each condition (i.e. BOLD responses explained by conditions of our GLM, not condition-irrelevant noise), the right entorhinal cortex represents affordances in our task.

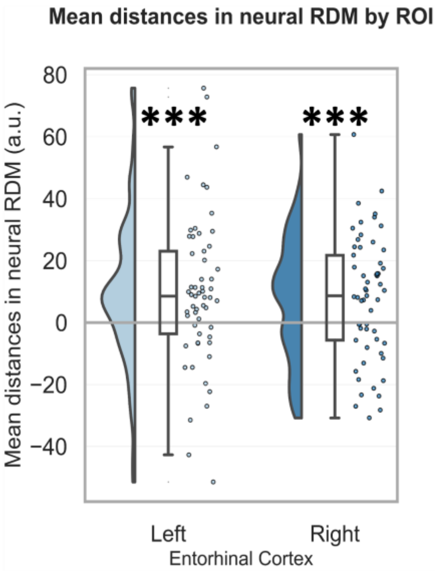

***S1 Appendix Fig A**: Cross-validated distance measures produce distance values greater than 0 if there are similar voxel patterns across runs. This allows testing for noise within specific ROIs. In both ROI used, we find mean distance values across all cells of the RDM matrix were greater than 0 (Wilcoxon signed-rank test, p<0.0001). All data and code underlying this figure are publicly available on Zenodo: https://doi.org/10.5281/zenodo.19209884*

Reliability-based voxel selection

To ensure that the main effect was not driven by noise at the voxel level, we implemented a modified version of reliability-based voxel selection (Tarhan & Konkle, 2020). The initial method was designed for condition-rich designs, and the authors advise caution in multivariate analyses due to potential imbalances. As the trials were balanced across conditions, we considered that the method was appropriate for RSA. Nonetheless, as the design had only four conditions, it was necessary to adapt the method to be sure that it was robust.

To do this, we considered a permutation-based approach. Rather than simply correlating betas in averaged halves of the data across conditions (as with the original method), which would involve a 4-by-4 correlation, we chose to (1) use Euclidean distance between vectors of beta values, which is more robust than correlation for small vectors, and (2) shuffle the data across conditions for a given voxel (1000 times), and re-calculate resultant distances to obtain an estimate of how likely a given Euclidean distance was based on the distribution of possible distances. Using this approach, we could assign a probability value to each voxel which described how much of the distribution was less than the actual, non-shuffled distance measure. A low probability value indicates a consistent response for a given voxel for each condition across runs.

The resultant probability maps were intersected with entorhinal ROIs to generate smaller ROIs within which voxels were more likely to be condition-responsive. Checks on simulated data confirmed that this procedure did not increase the rate of false positives. Nonetheless, this analysis assumes that the brain does indeed represent conditions differently, so it should be noted that results using this method should be interpreted as "[non] significant given that the brain region of interest is differentially responsive to the experimental conditions". As such, we consider these analyses confirmatory rather than a primary test of the main hypotheses.

As the probability values were typically quite high, we chose a liberal threshold of $p<0.2$ for the intersected ROIs (resulting in small ROIs: right entorhinal cortex mean voxels 31.2, std. 17.4; left entorhinal cortex mean voxels 22.3, std. 10.4). Despite the much smaller regions-of-interest, we still found a significant affordance effect in the right entorhinal cortex ($t(56)=2.30$; $p=0.0126$), suggesting the main finding is not the result of variations in voxel-level noise captured within anatomical regions-of-interest (left entorhinal cortex: affordance: $t(56)=0.75$; $p=0.227$).

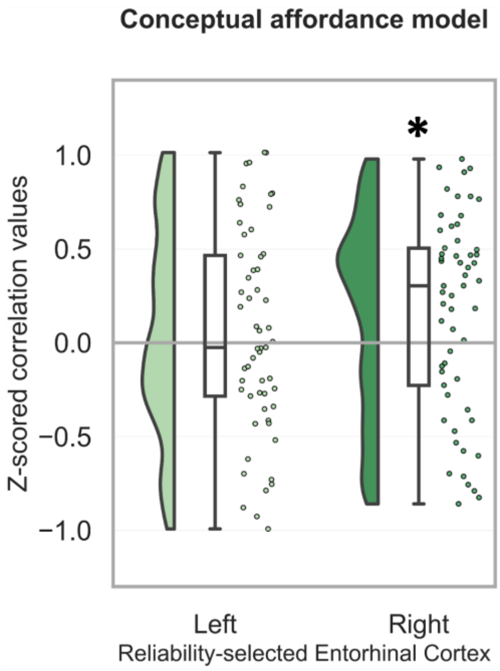

S1 Appendix Fig B*: Using a variant of reliability-based voxel selection, we created smaller regions-of-interest in which neural responses were more consistent, for each voxel, across all eight runs. Within these reduced ROIs, the main effects found in the primary analysis were still present, further confirming that our results are driven by signal rather than noise. All data and code underlying this figure are publicly available on Zenodo: https://doi.org/10.5281/zenodo.19209884*

Comparison of affordance effect in right entorhinal cortex to noise ceiling

To quantify the possible correlation of an ideal model with the right entorhinal cortical signal, we used inbuilt functions from rsatoolbox to quantify the noise ceiling (Nili et al., 2014; Schütt et al., 2023). The affordance model was not significantly below the noise ceiling (p=0.246). This indicates that the affordance effect was as strong as possible given experimental noise.

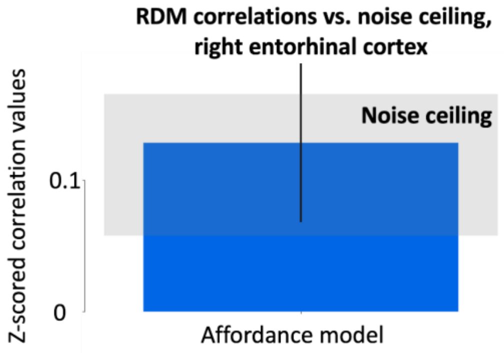

S1 Appendix Fig C*: The affordance model effect was within the noise ceiling (grey bar), indicating that the effect was as strong as possible given experimental noise and condition-*

wise responses. All data and code underlying this figure are publicly available on Zenodo: https://doi.org/10.5281/zenodo.19209884

Affordance effect in right entorhinal cortex is not driven by task difficulty

One possible confounding variable within our task could have been task difficulty, as there was a significant interaction between condition and reaction time (main Fig 2). To ensure that this was not the case, we ran a control analysis in which we regressed out a model based on average reaction time by state. The effect in the entorhinal cortex survived this correction (t(56)=2.079, p=0.0211).

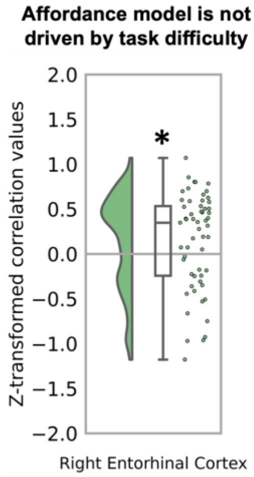

***S1 Appendix Fig D**: The affordance model effect still significantly explained neural pattern differences in the right entorhinal cortex even after accounting for a model of task difficulty based on reaction time. All data and code underlying this figure are publicly available on Zenodo: https://doi.org/10.5281/zenodo.19209884*

Distance in visual space correlates with visual but not entorhinal cortex

As our result was connected with gaze behaviour (main Fig 5), we checked if the affordance effect could be driven purely by bottom-up visual effects as the participants moved their eyes. Indeed, mapping of visual space has previously been demonstrated in the entorhinal cortex (Killian and Buffalo, 2018) and the entorhinal cortex has been proposed as a peak of the visual hierarchy (Fellemen and Van Essen, 1991). In this case, we would expect that more similar gaze positions would elicit more similar neural activity. This was tested using a model of state dissimilarity based on the Euclidean distance between the median positions in x and y across trials for each condition (see Methods for details). As with the control analyses above, differences in task timing and difficulty were accounted for via partial correlation of a reaction time model.

While gaze distances were represented in a visual cortex ROI (t(56)=2.119; p=0.0193), this was not the case in the right entorhinal cortex (t(56)=-0.771, p=0.778). Moreover, the effect

in the right entorhinal cortex is resilient to regressing out the visual model (t(56)=1.882, p=0.0369). This indicates that, although our results do show a connection between gaze behaviour and neural responses (main Fig 5), the affordance effect is not wholly explainable by basic visual effects.

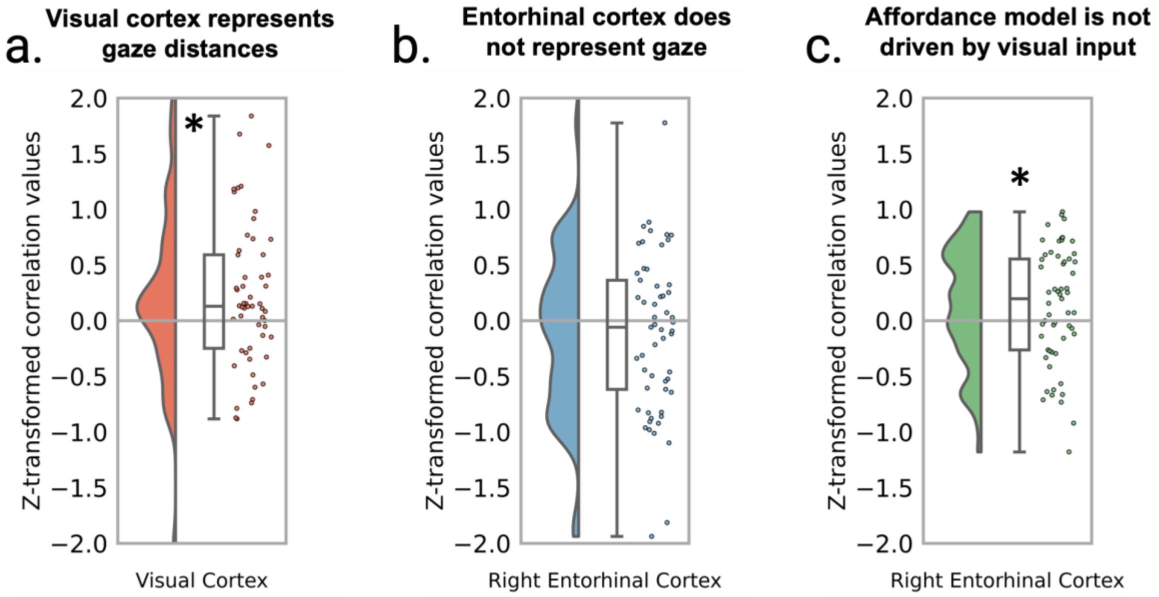

***S1 Appendix Fig E**: a and b. A model of gaze similarity correlates with neural pattern similarities in the visual cortex, but not the right entorhinal cortex. c. The effect in the right entorhinal cortex survived when this effect was controlled for in a partial correlation. All data and code underlying this figure are publicly available on Zenodo: https://doi.org/10.5281/zenodo.19209884*

Eye movement differences in the y-axis

Previous studies have shown an effect of numerical magnitude on eye movement in both the x and y axis (Viganò et al., 2024). We therefore tested eye movement effects in the vertical axis, following the same analysis procedure as before.

We found a significant difference in y-axis position at a cluster window of 1.191s to 1.622s (cluster permutation based on a two-tailed t-test; t(48)=4.06, p=0.012). However, unlike the effect in the x-axis there was no evidence for a correlation with task performance (r=0.116, p=0.442). Moreover, we were unable to replicate this result using estimated gaze position from the scanner session, using the same approach as on the x-axis whereby we compared the median position during a 1s window centred around the cluster identified on the first day across the two conditions (t(56)=-0.269, p=0.605).

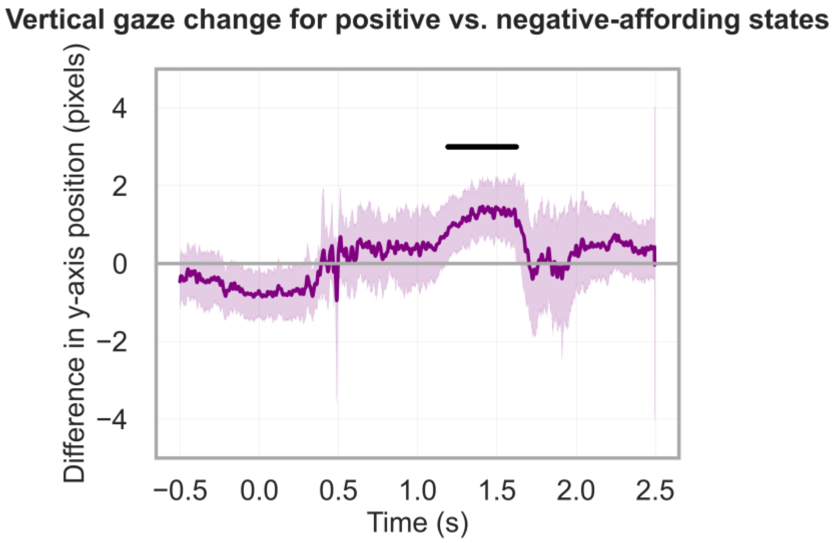

S1 Appendix Fig F. *Participants look further upwards for positive-affording states, relative to negative-affording states. All data and code underlying this figure are publicly available on Zenodo: https://doi.org/10.5281/zenodo.19209884*

Affordance representation in the motor cortex

It is possible that our task may have engaged somatosensory regions as a form of embodiment, congruent with the eye movement effect. As a post-hoc analysis, we tested the affordance model in the motor cortex separately in each hemisphere, using an ROI based on the conjunction of the 4a and 4p regions from the Juelich Atlas (3.0.3) maximum probability maps.

We found a similar pattern to the entorhinal cortex, whereby the left hemisphere does not represent affordances at a significance threshold of 0.025 (one-tailed, one-sided t-test against 0; $t(56)=-0.10$, $p=0.540$) while the right hemisphere does ($t(56)=2.22$, $p=0.0152$).

We should note that motor responses on probe trials were counterbalanced across conditions and used the right hand. Therefore, this effect likely reflects a genuine engagement of motor areas during our task rather than actual motor responses – although without EMG recordings we cannot exclude small movements we did not detect.

One interpretation could be a neural embodiment of action via the motor cortex, similar to the instantiation of action in eye movements. Motor cortical engagement without overt movement has previously been observed in fMRI studies during counting tasks (Tschentscher et al., 2012). If the left hand is typically used for counting smaller numbers, it is possible that thinking of small numerical operations (i.e. numbers from -2 to 2) may engage the right motor cortex.

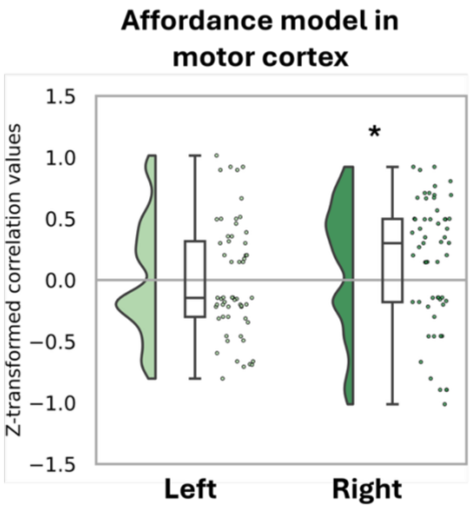

S1 Appendix Fig G. *Affordance representation in motor cortex. We found an effect of our affordance model in the right motor cortex, congruent with task embodiment. All data and code underlying this figure are publicly available on Zenodo: https://doi.org/10.5281/zenodo.19209884*

S1 Appendix References

Felleman, D. J., & Van Essen, D. C. (1991). Distributed Hierarchical Processing in the Primate Cerebral Cortex. Cerebral Cortex, 1(1), 1–47. https://doi.org/10.1093/cercor/1.1.1-a

Tschentscher, N., Hauk, O., Fischer, M. H., & Pulvermüller, F. (2012). You can count on the motor cortex: Finger counting habits modulate motor cortex activation evoked by numbers. NeuroImage, 59(4), 3139–3148. https://doi.org/10.1016/j.neuroimage.2011.11.037
