# Supplementary material for: Action information is integrated into entorhinal representations of conceptual space and is reflected in eye movements
Source: PLoS Biol. 2026 Apr 13;24(4):e3003755. doi: 10.1371/journal.pbio.3003755 (PMC13089867; doi:10.1371/journal.pbio.3003755)
Supplement: Attachment — Submitted filename: R2R_plosbio_v5.docx [file pbio.3003755.s003.docx]

We thank the reviewers for their time and attention in reviewing our manuscript. Below we address each review point by point.

In this updated version, we have addressed the additional point raised by Reviewer 3 suggesting the inclusion of null result in PhG and HPC in the main text. This has been noted below. Otherwise, this response is unchanged – we would like to take the opportunity to thank again the reviewers for their time and thoughtful comments during this review process.

**Reviewer #1:**

**Eperon et al. describe a well-controlled study of learning state transitions. Their focus is characterizing potential signatures of these state transitions in eye movement behaviour and neural representations in entorhinal cortex. Participants first learned hidden structure of transitions between numbers with some transitions forward or backward along the number line more likely than others in a behavioural and eye tracking session. On a second day, participants' recall of the state transitions was tested in a fMRI session. Results show that participants learned, that eye movements during training reflect shifts along a mental number line consistent with state transitions, and that neural representations in entorhinal cortex demonstrate pattern similarity consistent with state similarity. The authors argue that these findings suggest that entorhinal cortex represents actions in a conceptual space which are linked to eye movements.

This study offers compelling results with converging evidence from measures of behaviour, eye movements, and neural similarity. The methods employed are appropriate and well controlled. The statistical checks and supporting analyses are particularly noteworthy as they speak to the reliability of the reported effects. Overall, I find this work valuable and potentially an interesting contribution to the literature. However, several issues should be considered:**

Thank you for your time and detailed review. I have responded in-line to each comment below.

**1. To be honest, I found the abstract and introduction very confusing. There are many terms and jargon that are bandied about without properly defining them. And, there are many terms offered as equivalents that do not seem to match well. For example, "state transitions" are labelled "affordances", which itself is a very loaded term. Numerical operations are labelled as "actions". The authors refer to state transitions in the experiment's graph structure of numbers as "conceptual affordances". Of course, operational definitions that link constructs to measurable entities is the key to empirical design and the strength of any inference from findings is limited by the scope and appropriateness of operational definitions. In this case, there seems to be a disconnect between the very specific nature of the experiment (i.e., learning non-sequential transitions between numbers) and the targeted constructs. What is a conceptual affordance? And how well do state transitions of +/- 1
or 2 steps along a number line represent such an affordance? The title also feels like an overreach.**

We thank the reviewer for the careful evaluation and providing helpful suggestions to improve the manuscript. In response to this, we have revised the general manuscript text to aid readability, both in conceptual and the methodological aspects. This includes clearer definitions and more consistent use of terms throughout the manuscript.

Our work is based on the idea that the entorhinal cortex learns a state-action graph, using the RL sense of ‘action’ as ‘a function which causes a change in state’ (Sutton and Barto, 1998). The term ‘action’ is typically used for ‘motor action’ in cognitive neuroscience but is also used for *internal* action, such as changing one’s mind (Dayan, 2012; Metzinger, 2017). This is similar conceptually to ‘state transition’, but not the same: an action is the function which causes a transition between states. Therefore, we have clearly defined action and use this term more consistently, highlighting to the reader throughout that this is the intended meaning rather than using other terms.

Participants learn state-action associations: from each number, only two operations (actions) can be carried out from a set of four. We then aim to test if neural state similarities scale with the similarity of learned action associations for each state. The term ‘affordance’ derives from the need to communicate ‘the actions associated with a given state’. By analogy to the motor domain, we might say that a state ‘affords’ actions: although this use is clearly distinct from the use in ecological perception, others have used the term in both the context of learning relational structures (Behrens, 2018; Summerfield, 2021), navigation (Bonner and Epstein, 2017), and mental operations (McClelland, 2020). Therefore, we consider it a convenient way to communicate our main idea although we recognise that it is a term with a lot of theoretical ‘baggage’. In the interests of clarity, we have chosen to stick with the term ‘affordance’ but highlight more clearly that this is a distinct usage from that used in ecological perception. The term ‘conceptual affordance’ was intended to highlight this usage of ‘affordance’, but we recognise that it is not defined and adds jargon. Therefore, we have changed this to ‘affordance’. A discusssion can be found on page 2, using the following text:

In line with prior work on relational learning and memory, we use the term ‘action’ to refer to an operation which changes between states (Sutton & Barto, 1998; Dayan, 2012; Metzinger, 2017), and ‘affordance’ to refer to the set of actions associated with a state, with no necessary motor component (Bonner and Epstein, 2017; Behrens et al., 2018; McClelland, 2020). Importantly, the same action can be afforded (and performed) across different points of the space. That is, actions are factorised with respect to state identity and therefore can be learned and used in a state-independent manner (Dorrell et al., 2022; Bakermans et al., 2025). Here we focus on the entorhinal cortex, which has been proposed as a neural substrate for the abstraction and generalisation of task structure, including action information (Behrens et al., 2018). Previous work has shown that entorhinal cortex representations generalise task structure across different stimulus sets (Baram et al., 2021; Mark et al., 2024), and modelling work has suggested that the entorhinal cortex learns the actions afforded by these structures (Whittington et al., 2020), potentially as part of a larger system for compositional planning (Bakermans et al., 2025). Nonetheless, experimental evidence for this proposal is lacking. Therefore, we investigate if and how the entorhinal cortex represents action information within learned relational structures.

In terms of the operationalisation of states and actions as numbers and numerical operations, actions could be anything which relate different stimuli together as part of an interconnected graph. In this case, stimuli are linked in our task by mathematical operations. Therefore, we consider that our operationalisation is appropriate. Nonetheless, we agree that there is no experimental evidence here that our findings would generalise to other tasks or stimuli – the underlying theoretical work suggests that the entorhinal cortex would learn a state-action graph in any relational learning task (Whittington et al., 2020), but we only test one domain. We have tweaked the introduction and discussion to reflect this limited scope. However, given that the entorhinal cortex is responsive to conceptual information across a range of different stimuli (Bottini and Doeller, 2020), we consider that speculation about a more general role is warranted. We have been more specific through by referring to ‘a’ conceptual space, and discuss this explicitly on page 12 in the discussion:

Here we provide evidence that the human entorhinal cortex does represent actions in a conceptual space, and that these representations are linked to gaze behaviour.

We used a number line as base structure to which states with different afforded numerical operations (actions) were associated, thereby allowing us to distinguish action representations from other features of a state space (e.g. state identity, link distance). We find that the entorhinal cortex represents the actions 'afforded' by a given state in our design. It should be noted, however, that we only test a single conceptual domain: further work will be needed to establish to what extent action representation is domain-general, as suggested by models of the hippocampal formation (Whittington et al., 2022).

To reflect the above consideration and reduce jargon, we have changed the title to the following:

“Action in a conceptual space: representation of numerical operations in eye movements and the entorhinal cortex”

**2. Two aspects of the existing literature should be given more consideration in the manuscript. First, seminal work showing representations of conceptual spaces according to visual categories in hippocampus is omitted (e.g., studies by Zeithamova, Davis, and Mack) from the introduction and discussion. Second, the successor representation model is only noted in passing (e.g., Stachenfeld et al., 2017). This model is highly relevant to the current study and offers a compelling formal description of how state transitions based on reward/value are learned and encoded. It seems appropriate to give this account more consideration when connecting the current findings to the literature.**

We thank the reviewer for these suggestions. The particular focus on the entorhinal cortex (instead of hippocampus) and actions in the present manuscript caused us to originally overlook this indeed relevant literature, but we agree it should not be missed in the overview of relational processing in the hippocampal-entorhinal system. We have now included the work by Mack and Zeithamova on visual category learning in the respective paragraph of the introduction. The text can be found on page 2.

For instance, studies have observed that during transitions in two-dimensional feature spaces the entorhinal signal shows a hexa-directional modulation by the trajectory angle (Constantinescu et al., 2016; Viganò & Piazza, 2020, 2021; Park et al., 2021; Nitsch et al., 2023; Qasim et al., 2023; Schäfer et al., 2024; Viganò et al., 2024), mirroring the grid pattern observed during spatial navigation (Doeller et al., 2010). **In line with a role in navigation of feature spaces, the hippocampal formation appears to have a role in learning and updating of conceptual knowledge in the hippocampus (Mack et al., 2016, 2018; Zeithamova et al., 2019; Bowman et al., 2020). Moreover, hippocampal representations reflect underlying stimulus structure (Morton et al., 2020),** distances between positions in abstract spaces, such as link distance on a graph (Schapiro et al., 2012; Garvert et al., 2017, 2023; Pudhiyidath et al., 2021), distances in conceptual and social spaces (Theves et al., 2019, 2020; Park et al., 2021; Schafer et al., 2022), or location along a sound frequency spectrum (Aronov et al., 2017).

Furthermore, we added a new paragraph discussing successor representation models to the discussion on page 13. The text is as follows:

This is an intuition shared by models of cognitive maps (Behrens et al., 2018; Whittington et al., 2022), which have suggested that the entorhinal cortex either learns a state-action graph (Tolman-Eichenbaum Machine; Whittington et al., 2020) or is biased by the transition statistics of an environment, which are in turn subject to available actions (successor representation; Stachenfeld et al., 2017). Our observed action signal may be considered more in line with the first account, as successor representation models do not predict an explicit learning of action information in the entorhinal cortex. Nonetheless, our results may be compatible with distortions in transition structure as a function of different learned actions – indeed, previous fMRI work has shown representations of probabilistic task structure in the entorhinal cortex (Baram et al., 2021). As successor representation models are built around a predictive framework, future work may aim to test this by looking at how neural representations of action are related to trial-level choice behaviour (which we do not capture) or explicitly controlling reward distributions.

**3. The different states offer distinct transitions which means there's potentially interesting behavioural effects one might expect. As such, a breakdown of behavioural accuracy for each state would be helpful. Is there a difference in learning among the states? A more detailed look at learning across the states seems especially important in the context of understanding the neural similarity results. For example, if participants had better learning for states with higher conceptual affordance similarity, a further consideration of the RSA results may be warranted.**

This is an interesting point to consider. In the original manuscript, we tested for reaction time differences between states by comparing mean reaction times in probe trials, and found a difference between states (RM ANOVA, p<0.001). To control for this, we then carried out a control analyses in which mean reaction times per state were compared to create a ‘reaction time’ RDM. This was then excluded using a partial correlation (see Supplementary Figure 4).

We now carried out an additional analysis in which performance across states was compared during the scanner task. We calculated mean performance during probe trials for each state for every participant, and then repeated the previous analyses using a repeated measures ANOVA. This time, we did not find a significant effect of state identity on performance (F(3,168)=2.24, p=0.085). This has been added to the main text (see below). Therefore, we do not think that our neural results could be driven by performance differences. We note, moreover, that the general pattern of performance by state resembles RT differences (i.e. better performance for states 2 and 3 - see below), suggesting that our control analyses based on RT may capture potential sources of noise from task difficulty more generally.

A stimulus-independent encoding of the graph structure was supported by state-specific reaction time differences during probe trials. Reaction time data in the recall task on the second day showed a difference between response times for different states, comparing participant-wise mean reaction time for each state: state identity predicts reaction times (F(3, 168)=9.77, p<0.0001). Post-hoc pairwise comparisons show a significant difference between states 1 and 2 (t(56)=4.44, p<0.0001), states 1 and 3 (t(56=3.45, p=0.0011), states 2 and 4 (t(56)=-4.09, p=0.0001) and states 3 and 4 (t(56)=-3.38, p=0.0014). There were no significant differences between states 1 and 4 (t(56)=0.17, p=89) and states 2 and 3 (df(56)=-0.23, p=0.82). Despite these differences in reaction time across states, state identity did not significantly predict performance on probe trials, quantified as the percentage of correct responses following each state (F(3, 168)=2.24, p=0.085).

**4. One small point in the discussion: the authors propose that positional representations in entorhinal cortex may be updated by velocity input from eye movements. This is an interesting notion, but it does not seem to be well supported by the control analyses that showed there was no relationship between the distance of gaze position and entorhinal cortex representations. Of course, one cannot strongly argue from a null effect and the authors qualify their proposal with limitations. However, velocity would be reflected in moment-by-moment gaze position, so at least with the given data in the current study, this proposal lacks evidence.**

This is an interesting question – why do we find a (borderline) effect between eye movement and neural pattern distances for one state pair, but no effect of eye position?

We have toned down the paragraph and highlighted that this is speculation – given that our controls show no representation of gaze position, further work will be needed to establish if and how eye movement is connected with entorhinal representations. One key limitation in our case may be noise, as we did not use camera-based eyetracking.

One possible explanation is that the signal we see reflects a movement of attention. Covert attention (i.e. without eye movement) evokes navigation-like responses in EC (Giari et al., 2024), and has been proposed as a mechanism for navigating memory (Abrahamse et al., 2014; Viganò et al., 2024). This may explain our results: we would expect a link to eye movement in EC only when eye movement reflects attentional shifts, rather than for any change in eye movement.

The paragraph now reads as follows:

Based on our fMRI data, it is difficult to draw a direct link between action representation and specific neural populations. In light of the role for vision in primates and work suggesting a role of eye movements in ‘simulating’ conceptual navigation (Killian & Buffalo, 2018; Nau et al., 2018; Staudigl et al., 2018; Giari et al., 2023; Graichen et al., 2024; Viganò et al., 2023, 2024), it is conceivable that the observed eye movements reflect an embodiment of action. This could function as action input through cells with responses similar to saccade direction cells (Killian et al., 2015). At a more abstract level, eye movements could reflect movements of attention in the conceptual space (Ambrahamse et al., 2014; Giari et al., 2023; Viganò et al., 2024). If that is the case, shifts of internal spatial attention (rather than simple eye movements) could provide a velocity signal to the entorhinal system. This might explain why we find a tentative link between (estimated) eye movement and neural representations, but control analyses suggest that our entorhinal signal is not driven purely by eye movement (Supplementary Figure 5).

**5. Minor type: The text summary of learning performance references figure 1a, which should be figure 2a.**

Thank you, this has been corrected.

**Reviewer #2**

**Comments from the Academic Editor: Eperon and colleagues describe an fMRI and eye tracking study that measures representations of conceptual affordances in a task where human subjects transition between different 'states' using simple mathematical operations. The main prediction was that the entorhinal cortex would form an abstract representation of the possible transitions (affordances) between different states in a non-spatial task. There are two main findings: (1) eye tracking revealed that subjects tended to look left vs. right when possible affordances were backward on the number line vs forward on the number line, respectively, and (2) the (right) entorhinal cortex represented the affordances that were possible at a given number (state), as defined by representational similarity analyses.**

**The paper addresses an interesting topic and the predictions are well motivated. The paradigm and analyses are well thought out and the sample of subjects is quite large (over 50 subjects of fMRI data and over 40 for eye tracking). The main results are well motivated and are interesting. The paper is also generally well written and well organized. Supplementary results address some important questions (control analyses, etc.). Overall, my impression of the manuscript is quite positive and I believe the authors have put a lot of thought into the experiment and the writing. The two most significant weaknesses that I see are (1) the findings are somewhat incremental given other recent evidence of conceptual knowledge structures in the hippocampal formation, and (2) most of the core fMRI effects are "just barely" significant. That said, I do not view these as major weaknesses. With respect to the novelty, I do think the current experiment is different—in subtle, but interesting ways—from prior studies. With respect to the strength of findings, I think the results are very well motivated by prior studies (thus, there is somewhat of a 'prior') and I also think the control analyses presented as supplementary figures are very helpful (the control for reaction times is particularly important). Thus, I only have a couple of very minor suggestions.**

Thank you for your generous feedback and time spent reviewing the manuscript. I have responded to each comment in turn below.

**Major comments

1.      Given that there were very substantial reaction time differences between the different states, my biggest concern was that any RSA effects could simply reflect reaction time differences. Supplementary Figure 4 is therefore very important. However, it would be helpful to include a bit more information about exactly how this analysis was implemented. All that is said is that they ran "a control analysis in which we regressed out a model based on average reaction time by state." What was the reaction time model?  Was it a similarity matrix based on reaction time similarity?  What is reaction time similarity—just the raw difference in mean reaction times?  Were reaction times log transformed to correct for non-normality? Would that matter?   Again, the reaction time differences between conditions are very large and it certainly seems plausible that this could explain the results.**

We thank the reviewer for pointing this out and agree that further details are required. The reaction time model consisted of a participant-specific similarity matrix, in which states were compared based on the raw difference in mean reaction time across states. We extended the explanation in the revised manuscript.

To control for reaction time, the mean reaction time in probe trials across the entire fMRI session was taken within each subject and condition. The absolute difference in mean reaction time between states was used as a dissimilarity measure in order to construct a participant-specific representational dissimilarity matrix. This was then excluded via the partial correlation approach outlined above.

The raw difference was used rather than log-transformed reaction times in the initial control analysis. As the reviewer notes, log-tranformation might lead to a better approximation of normality. We therefore re-ran the analyses using the same approach but with log-transformed reaction times. the result changed (suggesting different ordering of RTs post log transformation), but the inference remains the same (t(56)=2.014, p=0.0244).

Importantly, we also find no significant differences in pairwise comparisons in the recation times of states and 2 and, which should be maximally different according to the affordance model (two-tailed paired t-test; df(56)=-0.23, p=0.82). This finding speaks against reaction times as an explanation of the affordance effect.

Additionally, we tested whether states differ in accuracy (i.e. correct or incorrect responses). We found no evidence for a difference between accuracy between states (F(3, 168)=2.24, p=0.085), further speaking against difficulty driving the affordance effect. This has been added to the main text on page 6 as follows.

Despite these differences in reaction time across states, state identity did not significantly predict performance on probe trials, quantified as the percentage of correct responses following each state (F(3, 168)=2.24, p=0.085).

**2.      I also wonder whether the RSA results observed in entorhinal might also be observed (perhaps more strongly) in motor cortex?  If so, that does not necessarily mean that the entorhinal results reflect task demands, but I think it would be relevant to test a motor region—for the same reason that it is useful to include visual cortex as a comparison in Supplementary Figure 5.**

We tested the affordance model in the motor cortex separately in each hemisphere, using an ROI based on the conjunction of the 4a and 4p regions from the Juelich Atlas (3.0.3) maximum probability maps.

We found a similar pattern to the entorhinal cortex, whereby the left hemisphere does not represent affordances at a significance threshold of 0.025 (one-tailed, one-sided t-test against 0; t(56)=-0.10, p=0.540) while the right hemisphere does (t(56)=2.22, p=0.0152).

It might be noted that the effect is not particularly strong considering that the motor cortex typically has a much lower noise level than the entorhinal cortex. Additionally, motor responses on probe trials were counterbalanced across conditions and used the right hand (which would typically elicit stronger responses in the left hemisphere). Therefore, this effect likely reflects a genuine engagement of motor areas during our task rather than actual motor responses – although without EMG recordings we cannot exclude small movements we did not detect.

One interpretation could be a neural embodiment of action via the motor cortex, similar to the instantiation of action in eye movements. Motor cortical engagement without overt movement has previously been observed in fMRI studies during counting tasks (Tschentscher et al., 2012). If the left hand is typically used for counting smaller numbers, it is possible that thinking of small numerical operations (i.e. numbers from -2 to 2) may engage the right motor cortex.

This result has been added to supplementary materials.

**Affordance representation in the motor cortex**

It is possible that our task may have engaged somatosensory regions as a form of embodiment, congruent with the eye movement effect. As a post-hoc analysis, we tested the affordance model in the motor cortex separately in each hemisphere, using an ROI based on the conjunction of the 4a and 4p regions from the Juelich Atlas (3.0.3) maximum probability maps.

We found a similar pattern to the entorhinal cortex, whereby the left hemisphere does not represent affordances at a significance threshold of 0.025 (one-tailed, one-sided t-test against 0; t(56)=-0.10, p=0.540) while the right hemisphere does (t(56)=2.22, p=0.0152).

We should note that motor responses on probe trials were counterbalanced across conditions and used the right hand. Therefore, this effect likely reflects a genuine engagement of motor areas during our task rather than actual motor responses – although without EMG recordings we cannot exclude small movements we did not detect.

One interpretation could be a neural embodiment of action via the motor cortex, similar to the instantiation of action in eye movements. Motor cortical engagement without overt movement has previously been observed in fMRI studies during counting tasks (Tschentscher et al., 2012). If the left hand is typically used for counting smaller numbers, it is possible that thinking of small numerical operations (i.e. numbers from -2 to 2) may engage the right motor cortex.

***Supplementary Figure 7.*** *Affordance representation in motor cortex. We found an effect of our affordance model in the right motor cortex, congruent with task embodiment.*

**3.      Ultimately, I was able to understand the experimental design, but it took a bit of time to figure it out.  I think it would be helpful to include a figure or text that very simply illustrates that state 1 = numbers that end in 1 or 6 (e.g., 21, 26),  and state 1 = +2 or -2, etc.   Again, I was able to figure this out, but it could have been easier.**

We have now added text to clarify the design, both to the methods (pages 15-18; see tracked changes) and results (page 4). the caption for Figure 1 has been extended to clearly explain how the states and numbers in 1c map together (page 5). We should note that state 1 does not always correspond to numbers ending in one, as the state-number assignment was randomised across participants to avoid possible visual confounds (i.e. one participant may have state 1 as 21, 26, and so forth, while another may have state 1 as 24, 29, etc.). This has been noted at the start of the results section using the following text.

We predicted that if the entorhinal cortex represents action in a conceptual space, then neural pattern similarities would scale with the action possibilities afforded by different states. We chose to operationalise states as numbers, and actions as numerical operations. This ensured a uniformity of action across different states.

In brief, the experiment consisted of two phases. Firstly, participants were taught a state-action graph in which numbers were associated with numerical operations. Secondly, the representation of individual numbers was probed using eyetracking (on the first day) and then fMRI (on the second day).

**Learning and generalisation of state-action associations**

Firstly, participants were taught a repeating graph consisting of states and actions projected on a number line. Specifically, they learned that certain numbers permitted transitions to other numbers, according to a predetermined graph structure consisting of repeating modules (see Figure 1c). They learned this through free exploration along the graph using button presses (Figure 1b). Each of the four states afforded a certain subset of actions: state 1 afforded -2 or +2, state 2 afforded +1 or +2, state 3 afforded -2 or -1, and state 4 afforded -2 or +2. In the task, therefore, if the number ‘49’ was mapped onto state 2, participants would be given a choice of changing to numbers ‘50’ or ’51’ via a left or right button press. Number-state mappings varied across participants by shifting the graph structure along the number line. Therefore, while one participant learned that ‘49’ (state 2) could transform into ’50’ or ‘51’, another learned that ‘50’ (state 2) could transform into ‘51’ or ‘52’. These constituted a form of non-spatial affordance: each number was associated with a specific subset of actions based on state assignment.

**4.      Relatedly: there is a line in the text that says "In particular, eye movements could be expected to skew more rightwards for states that allow only positive actions (e.g. state 2) relative to states that allow only negative actions (e.g. state 3)."   The use of "e.g." (for example) is confusing because it implies that there are OTHER states that also allow for only positive actions or only negative actions. But (unless I missed something), it is ONLY state 2 and state 3 that are relevant for this analysis. Thus, it should be "i.e.," instead of "e.g."   It's a minor point, but it created some confusion for me.**

Good spot, this has been changed.


 **Reviewer #3:**

**Summary
The authors report results from fMRI and eye tracking experiments (57 and 46 human subjects, respectively). They describe that fMRI voxel patterns in the right entorhinal cortex correlate with similarities of possible actions in a task with mathematical operations. Gaze was correlated with positive versus negative mathematical operations. The authors suggest that these findings indicate that action information is integrated into entorhinal representations of conceptual spaces. This is an interesting manuscript with a range of analyses, which would benefit from a clearer presentation with important information included earlier in the main text. Please find my comments listed below.**

Thank you for your time in reviewing the manuscript. We have addressed each comment in line below.
 **Major comments
(1) The introduction and discussion talk about the involvement of the hippocampal formation in mapping relational knowledge, but fMRI RSA focuses on the entorhinal cortex. The rationale for just looking at the entorhinal cortex is unclear. Several MTL regions should be tested to establish the specificity of the effects to the entorhinal cortex.**

We thank the reviewer for pointing out this conceptual unclarity, which we agree needs to be better specified. The rationale for addressing the entorhinal cortex specifically is based upon theories which propose the entorhinal cortex as a region which learns a state-action graph. This has been more clearly stated in the introduction, as the specificity of our prediction was not immediately obvious in the previous version of the manuscript. The text is as follows:

Here we focus on the entorhinal cortex, which has been proposed as a neural substrate for the abstraction and generalisation of task structure, including action information (Behrens et al., 2018). Previous work has shown that entorhinal cortex representations generalise task structure across different stimulus sets (Baram et al., 2022; Mark et al., 2024), and modelling work has suggested that the entorhinal cortex learns the possible actions within these structures (Whittington et al., 2020), potentially as part of a larger system for compositional planning (Bakermans et al., 2024). Nonetheless, experimental evidence for this proposal is lacking. Therefore, we investigate if and how the entorhinal cortex represents action information within learned relational structures.

Nonetheless, it is an interesting question to what extent our effect is limited to the entorhinal cortex. To test this, we repeated the ROI analysis (bilaterally) in other MTL regions, namely the hippocampus and parahippocampal gyrus. As before, ROIs were drawn from the Juelich Atlas maximum probability maps, converted to subject space and intersected with a probabilistic grey matter mask thresholded at 0.5.

We found no significant group-level correlations between neural patterns in these regions and our model of interest (affordances). In the right hippocampus we observed a trend in the same direction as in the EC. All results are drawn from a one-sided, one-tailed t-test against 0 using a significance threshold of 0.025 to match the entorhinal results.

Hippocampus left: t(56)=1.238, p=0.110

Hippocampus right: t(56)=1.587, p=0.059

Parahippocampal gyrus left: t(56)=-1.011, p=0.842

Parahippocampal gyrus right: t(56)=0.268, p=0.395

We have now reported these analyses in the main text of the manuscript, using the following text on page 8:

As a further control analysis to check for the specificity of the effect within the medial temporal lobes, we also tested our affordance model in the hippocampus and parahippocampal gyrus, controlling for link distance and affordance magnitude. We found no evidence for an affordance representation in these regions (hippocampus left: t(56)=1.24, p=0.11; hippocampus right: t(56)=1.59, p=0.059; parahippocampal gyrus left: t(56)=-1.01, p=0.84; parahippocampal gyrus right: t(56)=0.27, p=040).

We have also added details on ROI selection for these regions to the methods.

**(2) The task should be better explained in the main text, and the different states should be explained early on. For example, in Fig. 1a, which of the numbers correspond to states 1, 2, 3, and 4 (same for Fig. 1c)? Also, in Fig. 1c, what does S1-4 mean? When the recall task is first mentioned in the main text, it is unclear what it refers to, etc. This opaqueness in task description makes it hard to follow the rest of the results.**

We have added additional explanation of the task at the start of the results section, and added details to figure labels explaining what the states mean. In general, we have cleaned up the text to make terminology and task design clearer. Changes have been made throughout, but most substantially on page 4, as follows.

We predicted that if the entorhinal cortex represents action in a conceptual space, then neural pattern similarities would scale with the action possibilities afforded by different states. We chose to operationalise states as numbers, and actions as numerical operations. This ensured a uniformity of action across different states.

In brief, the experiment consisted of two phases. Firstly, participants were taught a state-action graph in which numbers were associated with numerical operations. Secondly, the representation of individual numbers was probed using eyetracking (on the first day) and then fMRI (on the second day).

Learning and generalisation of state-action associations

Firstly, participants were taught a repeating graph consisting of states and actions projected on a number line. Specifically, they learned that certain numbers permitted transitions to other numbers, according to a predetermined graph structure consisting of repeating modules (see Figure 1c). They learned this through free exploration along the graph using button presses (Figure 1b). Each of the four states afforded a certain subset of actions: state 1 afforded -2 or +2, state 2 afforded +1 or +2, state 3 afforded -2 or -1, and state 4 afforded -2 or +2. In the task, therefore, if the number ‘49’ was mapped onto state 2, participants would be given a choice of changing to numbers ‘50’ or ’51’ via a left or right button press. Number-state mappings varied across participants by shifting the graph structure along the number line. Therefore, while one participant learned that ‘49’ (state 2) could transform into ’50’ or ‘51’, another learned that ‘50’ (state 2) could transform into ‘51’ or ‘52’. These constituted a form of non-spatial affordance: each number was associated with a specific subset of actions based on state assignment.

Knowledge of the graph was tested using blocked test trials presenting two candidate successors, of which only one corresponded to a possible transition and had to be chosen (Figure 1b top right). This tested if participants had correctly learned the afforded actions.

Participants were trained on two consecutive days to learn how specific numbers could 'transform' into other numbers. By the end of the first day, participants were able to reliably select the correct transitions for different numbers (accuracy: 84.7%; chance level 50%, std. 7.1%, last ten blocks; figure 2a). Moreover, they were able to apply the learned transformations to new numbers: after training on the second day, they reached a performance level of 88.8% on unseen numbers (std. 11.3%). The application of these operations to hitherto-unseen numbers indicates that participants understood that numbers and transitions were factorised.

In two subsequent sessions using the eyetracker and in the MRI scanner, respectively, participants were then presented with individual numbers in a pseudorandom order. In these sessions their knowledge of the graph was probed using probe trials showing two numbers, to which participants had to respond if both were the correct successors for the previously presented number or not (Figure 1b bottom). This task was designed to extract a representation of neural or ocular signal for each number, which would then be used to test our main hypothesis.

**(3) Why did you apply slice time correction during fMRI preprocessing given that your TR was 1s?**

It is correct that smaller TRs will reduce the relative importance of slice time correction. Nonetheless, the timecourse of slice acquisition means that slice time correction is still relevant to ensure that the image is centred around the correct timepoint, especially in faster designs such as ours. This is the default setting used by fmriprep, which we used in order to ensure replicability.

**(4) Why didn't you extract subject-specific ROIs using FreeSurfer? The masks from the Jülich atlas may not closely correspond to the subject's individual anatomy.**

Subject-specific masks were generated by transforming the Juelich atlas (in MNI space) to subject space, using the transformation generated by fmriprep during preprocessing. They were then intersected with a probabilistic grey matter mask from fmriprep, thresholded at 0.5.

The Juelich atlas is widely used in our field due to probabilistic mapping of region boundaries based on histology. In our case one advantage was the availability of ‘maximum probability maps’, which ensure that voxels are more reliably assigned to the EC than other regions of the MTL. As our hypothesis targeted the EC, we wanted to be sure that ROI selection was not suscepible to variations in surface mapping (from Freesurfer) which might lead to taking into account voxels from other MTL regions.

We acknowledge that there may be individual variation due to subject anatomy, as the normalisation may not always map perfectly onto the gyri and sulchi of the brain. Nonetheless, our ROI choice reflects a trade-off between individual anatomy and population-level EC definition.

In our opinion, the ideal approach would have been to run a functional localiser for ROI definition, but there is unfortunately no task which would reliably isolate the EC at an individual level.

**(5) The unsignificant whole-brain RSA should be reported in the main text.**

This has been added to the main text on page 9, using the following text.

Next, we ran a whole-brain searchlight to establish the spatial distribution of the affordance effect. For each participant, each voxel was taken as the centre of a sphere, which was used to run representational similarity analysis as before. Whole brain maps were then transformed to MNI space, and one-sided t-tests were used to compare group-level correlation scores at each voxel to zero. However, no voxels survived cluster correction (threshold-free cluster enhancement; all p>0.1).

**(6) It was unclear to me whether it is justified to equate actions, possible transitions, and affordances.**

We define ‘action’ as a mechanism which changes state, and ‘affordance’ as the range of possible actions available at each state. This is in line with previous uses in reinforcement learning and cognitive maps (Behrens et al., 2018), although we recognise that ‘action’ and ‘affordance’ are more frequently used with reference to motor action and ecological perception. Therefore, we have made the definitions more explicit throughout the text, and revisited terminology to ensure consistent usage (page 2, and throughout).

The use of ‘possible transition’ is perhaps confusing, as this is distinct from the action itself: a participant may be able to transition from 2 to 4, but the action itself is +2. Initially, this term was used as it might be considered more accessible, but in retrospect it may overcomplicate ther terminology used. To avoid extra jargon, we have removed this term from the paper.

Our terminology has been clarified on page 2, using the following:

In line with prior work on relational learning and memory, we use the term ‘action’ to refer to an operation which changes between states (Sutton & Barto, 1998; Dayan, 2012; Metzinger, 2017), and ‘affordance’ to refer to the set of actions associated with a state, with no necessary motor component (Bonner and Epstein, 2017; Behrens et al., 2018; McClelland, 2020). Importantly, the same action can be afforded (and performed) across different points of the space. That is, actions are factorized with respect to state identity and therefore can be learned and used in a state-independent manner (Dorrell et al., 2022; Bakermans et al., 2025). Here we focus on the entorhinal cortex, which has been proposed as a neural substrate for the abstraction and generalisation of task structure, including action information (Behrens et al., 2018). Previous work has shown that entorhinal cortex representations generalise task structure across different stimulus sets (Baram et al., 2021; Mark et al., 2024), and modelling work has suggested that the entorhinal cortex learns the actions afforded by these structures (Whittington et al., 2020), potentially as part of a larger system for compositional planning (Bakermans et al., 2025). Nonetheless, experimental evidence for this proposal is lacking. Therefore, we investigate if and how the entorhinal cortex represents action information within learned relational structures.

**(7) It was also unclear to me whether the term "numerical operations" should be generalized to "actions," as the latter encompasses far more than numerical processes. Moreover, since German students are typically familiar with the concept of a number line, the observed effects may be biased by this prior knowledge.**

This question regards operationalisation of our main hypothesis. I will first address prior knowledge. The number-state assignment was balanced across participants (so that some would have learned that 21, 26, 31 etc. afford +1 and +2, whereas others would have learned this for 22,27,32 etc.). Therefore, any existing knowledge relating to individual numbers could not explain our findings. This has been clarified in the main text. In terms of knowledge of the number line, this is a feature rather than a bug which ensures uniformity of action representation: the number line ensures that participants understand the same action takes you between 22 and 24 as 67 and 69 (for example). This has also been noted in the results section on page 4.

‘Action’ is indeed a much wider definition, and numerical cognition is a specific field in its own right. More work will be needed to establish if this effect is true across multiple domains, and we have added this consideration to the discussion. Nonetheless, as prior studies have shown entorhinal responses in multiple conceptual domains (Bottini and Doeller, 2020), we believe speculation about a more general role is warranted. We have added more qualification to the discussion to make clear that we are talking about a single conceptual space, without experimental evidence for a domain-general representation of action beyond numbers (page 12). Moreover, the title has been modified to the following to reflect that we test only a single domain:

“Action in a conceptual space: representation of numerical operations in eye movements and the entorhinal cortex”

**(8) The authors seem to use the terms cognitive maps (introduction) and cognitive graphs (task description) interchangeably, but I do not believe that this is correct (e.g., Peer et al., TiCS, 2021).**

In the main text, we refer both to ‘cognitive maps’ and to ‘graphs’. According to some theories, a cognitive map could be built around a learned graph (Whittington et al., 2022; Stachenfeld et al., 2017) rather than a continuous, map-like format. Peer (2021) presents alternative terminology of a ‘cognitive graph’ to express this idea, but it should be noted that ‘cognitive map’ has historically been used for either representational format (Behrens et al., 2018).

In our case, we have no experimental data to suggest that the underlying format is either map-like or graph-like. The experiment itself is built around a state-action graph, but this could nonetheless be projected onto a continuous space. We use the term ‘graph’ in this context, to refer to the task structure rather than a specific theory of representational format.

We have revised our terminology where appropriate to highlight relations in general rather than a specific format and highlighted that both graphs and continuous spaces can be seen as the underlying structure of a cognitive map (page 2).

Accordingly, different models of cognitive maps integrate action input as the cue to move between different states of learned graphs (Stachenfeld et al., 2017; Whittington et al., 2020; George et al., 2021) or a pre-structured Euclidean space (Chandra et al., 2025).

**Minor comments
(1) Why did you use Mahalanobis distance for estimating the RDMs? To the best of my knowledge, Pearson correlations are standard. Please explain and provide the results from Pearson correlations.**

Cross-validated mahalanobis (crossnobis) distance is the recommended neural distance from the authors of rsatoolbox due to increased reliability and less bias (Walther et al., 2015). It has major benefits in noise reduction, from taking into account both the shape of the noise distribution (as estimated from residuals during Mahalanobis distance) and consistency across experimental runs (through cross-validation). In our case, this provides substantial advantages due to the high noise level of the entorhinal cortex relative to other brain regions.

As Pearson is also widely used in the community (despite increased noise levels and an assumption of linearity between multivariate patterns), we re-ran the main analysis using Pearson correlation for RDM estimation. The effect was reduced to just below significance at the threshold of 0.025 (t(56)= 1.94, p=0.0287), but the trend is maintained.

**(2) Was there also a difference in eye tracking y-position between states 2 and 3?**

We tested this and found that there was indeed a significant difference in the y-axis. Unlike the x-axis effect, however, this was not correlated with performance and was not replicated in the fMRI analyses using deepMReye. This has been reported in the Supplementary Materials as follows:

**Eye movement differences in the y-axis**

Previous studies have shown an effect of numerical magnitude on eye movement in both the x and y axis (Viganò et al., 2024). We therefore tested eye movement effects in the vertical axis, following the same analysis procedure as before.

We found a significant difference in y-axis position at a cluster window of 1.191s to 1.622s (cluster permutation based on a two-tailed t-test; t(48)=4.06, p=0.012). However, unlike the effect in the x-axis there was no evidence for a correlation with task performance (r=0.116, p=0.442). Moreover, we were unable to replicate this result using estimated gaze position from the scanner session, using the same approach as on the x-axis whereby we compared the median position during a 1s window centred around the cluster identified on the first day across the two conditions (t(56)=-0.269, p=0.605).

***Supplementary Figure 6.*** *Participants look further upwards for positive-affording states, relative to negative-affording states.*

**(3) Link distance and action magnitude should be explained in the main text.**

An explanation for these models has been added to the results section (page 8).

**(4) "'2' to '5' would be similar to '55' to '57'" seems wrong.**

True, corrected – thank you.

**(5) "median change in gaze position across every timepoint within all previously-identified significant clusters" - were there multiple significant clusters? Fig. 3b shows only one cluster.**

There was only one significant cluster – this phrasing reflected the code implementation of taking all significant timepoints. We have changed this to specify that we are talking about a single cluster.

**(6) "which would involve a 4-by-4 correlation" - why is it a 4-by-4 correlation? Aren't six values correlated with each other?**

This phrase comes from the Supplementary Materials, where we are describing the method used for reliability-based voxel selection. In this case, the correlation refers to taking voxel responses in two halves of the dataset (runs 1-4 and runs 5-8), averaging across runs for each condition, and correlating activation values for each voxel across these two subsets in order to see how consistently this voxel responds to the same experimental condition. We have four conditions, as we have four states. Therefore, this is a 4-by-4 correlation (unlike the RSA, which is indeed a 6-by-6 correlation as the conditions are compared across each other).

Our own method gets around this by using a permutation based on Euclidean distances. We have taken this opportunity to extend and explain our method better in the supplementary materials using the subsequent text.

**Reliability-based voxel selection**

To ensure that the main effect was not driven by noise at the voxel level, we implemented a modified version of reliability-based voxel selection (Tarhan & Konkle, 2020). The initial method was designed for condition-rich designs, and the authors advise caution in multivariate analyses due to potential imbalances. As the trials were balanced across conditions, we considered that the method was appropriate for RSA. Nonetheless, as the design had only four conditions, it was necessary to adapt the method to be sure that it was robust.

To do this, we considered a permutation-based approach. Rather than simply correlating betas in averaged halves of the data across conditions (as with the original method), which would involve a 4-by-4 correlation, we chose to (1) use Euclidean distance between vectors of beta values, which is more robust than correlation for small vectors, and (2) shuffle the data across conditions for a given voxel (1000 times), and re-calculate resultant distances to obtain an estimate of how likely a given Euclidean distance was based on the distribution of possible distances. Using this approach, we could assign a probability value to each voxel which described how much of the distribution was less than the actual, non-shuffled distance measure. A low probability value indicates a consistent response for a given voxel for each condition across runs.

The resultant probability maps were intersected with entorhinal ROIs to generate smaller ROIs within which voxels were more likely to be condition-responsive. Checks on simulated data confirmed that this procedure did not increase the rate of false positives. Nonetheless, this analysis assumes that the brain does indeed represent conditions differently, so it should be noted that results using this method should be interpreted as "[non] significant given that the brain region of interest is differentially responsive to the experimental conditions". As such, we consider these analyses confirmatory rather than a primary test of the main hypotheses.

As the probability values were typically quite high, we chose a liberal threshold of p<0.2 for the intersected ROIs (resulting in small ROIs: right entorhinal cortex mean voxels 31.2, std. 17.4; left entorhinal cortex mean voxels 22.3, std. 10.4). Despite the much smaller regions-of-interest, we still found a significant affordance effect in the right entorhinal cortex (t(56)=2.30; p=0.0126), suggesting the main finding is not the result of variations in voxel-level noise captured within anatomical regions-of-interest (left entorhinal cortex: affordance: t(56)=0.75; p=0.227).
